# Selected Essays and Addresses by Sir James Paget

**Published:** 1902-09

**Authors:** 


**Selected Essays and Addresses by Sir James Paget.** Edited by STEPHEN PAGET. Pp. viii., 445. London: Longmans, Green and Co. 1902.

This collection of essays is intended to be "welcome to men who knew him." His earlier lectures are not included, but the selection is such as any one of his pupils might wish to have in honour of him and for love of him. They were given on various occasions, the most noteworthy perhaps being the presidential address at the opening of the International Medical Congress of 1881, which made a profound impression on the large and brilliant audience presided over by the Prince of Wales. None of the essays can be considered out of date; the subjects are for the most part perennial in their nature, and the writer's views would to the last have been the same as when he first penned them. Anything written by such a master of the English language cannot fail to be of lasting service. It is gratifying to us to observe what is said of Paget by Da Costa, who writes that "he spoke frequently and always well. . . . He could speak impromptu with a grace and a beauty which charmed his audience. His lectures were intellectual treats. . . . His more finished addresses . . . were delivered with such ease that they gave the impression of unstudied force rather than of careful elaboration." He being dead yet speaketh.

